# Anxiolytic Effects of *Cichorium intybus* L. Oligo-Polysaccharides by Modulating Gut Microbiota, Neuronal Signaling Pathways, and Neuroinflammation in Chronic Sleep Deprivation-Stressed Mice

**DOI:** 10.3390/foods14111859

**Published:** 2025-05-23

**Authors:** Yongzhi Zhao, Yiwen Zhang, Yanqin Luo, Fang Chen, Meng Qiang, Mengchao Liu, Ruile Pan, Qi Chang, Ning Wang, Muhammad Wasim Usmani, Ning Jiang, Xinmin Liu

**Affiliations:** 1 Institute of Drug Discovery Technology, Ningbo University, Ningbo 315211, China; 2 Qian Xuesen Collaborative Research Center of Astrochemistry and Space Life Sciences, Ningbo University, Ningbo 315211, China; 3 Research Center for Pharmacology and Toxicology, Institute of Medicinal Plant Development (IMPLAD), Chinese Academy of Medical Sciences and Peking Union Medical College, Beijing 100193, China; 4 Key Laboratory of Ministry of Education for Xinjiang Phytomedicine Resource and Utilization, School of Pharmacy, Shihezi University, Shihezi 832003, China

**Keywords:** chronic sleep deprivation, anxiety-like behavior, gut microbiota, 16S rRNA sequencing, neuroinflammation, BDNF-P13K/AKT/mTOR pathway

## Abstract

Prolonged sleep deprivation impairs brain function and increases the risk of mental health disorders. *Cichorium intybus* L. Oligo-polysaccharides (JSO), bioactive compounds derived from chicory, belong to the category of food-medicine homologous substances, possess gut microbiota-modulating and anti-inflammatory properties, and serve as a natural prebiotic, having significant research value in food science. This research examined the anxiolytic properties of JSO in a murine model subjected to chronic sleep deprivation (CSD) stress and explored the mechanisms behind this effect, providing experimental evidence for the development of *Cichorium intybus* L. as a functional food. Specific pathogen-free (SPF) KM male mice were allocated at random into six experimental groups: the control group, the CSD model group, the diazepam (10 mg/kg) group, and the JSO treatment groups at low (50 mg/kg), medium (100 mg/kg), and high (200 mg/kg) doses. Following 3 weeks of CSD, anxiety-like behaviors were assessed using the open field test, elevated plus maze test, light–dark box test, forced swim test, and marble-burying test. To analyze the composition of gut microbiota, 16S rRNA sequencing was employed, while protein expression in the BDNF, PI3K/AKT/mTOR, and NLRP3 inflammasome pathways was detected by Western blot. Behavioral analysis indicated that JSO (at doses of 100 and 200 mg/kg) markedly enhanced both the time allocated to open arms and the number of entries into open arms in the elevated plus maze test (*p* < 0.05). JSO (at doses of 50 and 200 mg/kg) significantly elevated transitions in the light–dark box test (*p* < 0.05), all JSO doses drastically cut down marble-burying behavior (*p* < 0.001, *p* < 0.01, *p* < 0.01). The 16S rRNA sequencing indicated that JSO intervention increased Bacteroidetes abundance while reducing Actinobacteria. Western blot analysis demonstrated that JSO significantly downregulated the ratios of p-mTOR/mTOR, p-PI3K/PI3K, p-AKT/AKT, BAX/BCL-2, as well as the expression levels of NLRP3, ASC, Caspase-1, and IL-6 proteins (*p* < 0.05), while upregulating hippocampal BDNF (*p* < 0.05). These results indicate that JSO ameliorates CSD-induced anxiety-like behaviors by restoring gut microbiota homeostasis, regulating the BDNF-PI3K/AKT/mTOR and BAX/BCL-2 apoptosis pathways, and suppressing NLRP3 inflammasome-mediated neuroinflammation.

## 1. Introduction

Anxiety disorders represent a major challenge to mental health globally, imposing significant burdens on individuals as well as the larger community [1,2]. Beyond psychological distress, anxiety disorders are associated with various physiological complications, including cardiovascular diseases and immune system dysfunction [3,4]. Current pharmacological treatments for anxiety primarily include benzodiazepines (such as diazepam) and selective serotonin reuptake inhibitors (SSRIs, like fluoxetine). However, these medications have notable limitations. Benzodiazepines can lead to dependence and tolerance, with long-term use increasing the risk of cognitive impairment [5,6,7]. SSRIs, while effective for many patients, may cause gastrointestinal disturbances, sexual dysfunction, and other adverse effects [8,9]. Moreover, a subset of patients exhibits inadequate responses to existing pharmacotherapies, underscoring the need for novel therapeutic alternatives. Lately, natural compounds and their bioactive components have gained attention in anxiety research due to their multi-target and favorable safety profiles [10,11]. Identifying and developing safe, effective, and well-tolerated anti-anxiety agents is therefore of significant clinical and societal importance.

*Cichorium intybus* L., a perennial Asteraceae species native to Europe, now grows widely across Asia, the Americas, and Oceania. In China, it primarily grows in the northwest, northeast, and north-central regions [12]. Traditionally, chicory has been used in Uygur and Mongolian medicine for its therapeutic properties, including heat-clearing, detoxification, diuresis, anti-edema, and digestive benefits [13]. The bioactive constituents of *Cichorium intybus* L. include oligo-polysaccharides, flavonoids, phenolic acids, coumarins, and terpenoids. Among these, chicory polysaccharides have demonstrated diverse pharmacological effects, such as antihypertensive and lipid-lowering properties, glycemic and insulin regulation, immunomodulation, anti-tumor activity, and intestinal inflammation alleviation [14,15,16]. Additionally, chicory oligosaccharides and polysaccharides selectively stimulate the growth of advantageous gut bacteria, including *Bifidobacterium* and *Lactobacillus*, while pathogenic bacteria (e.g., *Clostridium*) are suppressed. They also enhance the production of short-chain fatty acids, thereby contributing to gut microbiota hemostasis and functioning as natural prebiotics [17]. Furthermore, chicory exhibits antioxidant, anti-aging, and mineral absorption-enhancing properties. The anti-anxiety effects of *Cichorium intybus* L. Oligo-polysaccharides were demonstrated in our previous studies [18,19], However, research on their precise mechanisms in alleviating anxiety-like behaviors induced by chronic stress remains limited. Further investigation is required to clarify the potential mechanisms underlying the anxiolytic effects of JSO.

The chronic sleep deprivation (CSD)-induced anxiety model is a critical tool for investigating the physiological, behavioral, and neurochemical consequences of sleep loss. Models of sleep deprivation are commonly utilized to explore the mechanisms that lead to the onset and progression of anxiety disorders, in addition to evaluating the efficacy of pharmacological interventions in delivering protection [20]. In this research, an anxiety model in mice was established via the CSD paradigm (rotating drum method), followed by intervention with JSO. This study investigated the mechanisms underlying JSO’s anxiolytic effects by examining gut microbiota composition, apoptosis, and neuroinflammation. The results support JSO’s potential as a novel therapeutic candidate for anxiety disorders.

## 2. Materials and Methods

### 2.1. Drugs and Reagents

The *Cichorium intybus* L. Oligo-polysaccharides (JSO) were purchased from Torlin Pharmaceutical Co., Ltd. (Batch No.: YF230202, purity: 75.25%, Qingdao, China); Diazepam was supplied by Shandong Xinyi Pharmaceutical Co., Ltd. (Batch No.: H37023039, Shandong, China); The BCA Protein Assay Kit was provided by Solarbio Science & Technology Co., Ltd. (Beijing, China, BC3710); The antibodies that were used in this study were as follows: p-mTOR (CST, Danvers, MA, USA, 5536S); mTOR (Abcam, Cambridge, UK, ab32028); p-PI3K (Abcam, Cambridge, UK, ab17478S); PI3K (CST, Danvers, MA, USA, 24292); p-AKT and AKT (CST, Danvers, MA, USA, 4060S/4685S); BDNF (Abcam, Cambridge, UK, ab108319); p-NF-κB (Abcam, Cambridge, UK, ab131100); NF-κB (CST, Danvers, MA, USA, 8242); BAX and BCL-2 (Abcam, Cambridge, UK, ab32503/ab194583); NLRP3 (Abcam, Cambridge, UK, ab263889); ASC (Abcam, Cambridge, UK, ab180799); Caspase-1 (ABclonal Technology, Wuhan, China 67314); and IL-6 (Abcam, Cambridge, UK, ab9324).

### 2.2. Experimental Design and Ethical Approval

A total of 72 male KM mice, classified as specific pathogen-free and aged between 4 and 5 weeks with weights ranging from 24 to 27 g, were acquired from Vital River Laboratory Animal Technology Co., Ltd. based in Beijing, China [SCXK (Jing) 2021-0006]. These mice were kept at the Institute of Medicinal Plant Development, Chinese Academy of Medical Sciences [SYXK (Jing) 2023-0008], under carefully regulated environmental parameters: a temperature range of 22–24 °C, relative humidity of (55% ± 10%), along with a 12 h light/dark cycle. Water and food were available at all times. After a week’s acclimatization, mice were allocated at random into six groups: (1) the control group, (2) the CSD model group, (3) the diazepam group (10 mg/kg), and (4–6) the JSO treatment groups at low (50 mg/kg), medium (100 mg/kg), and high (200 mg/kg) doses (each group, *n* = 12). Throughout the experimental period, all mice received treatments via oral gavage. The drug administration design for the experimental group was as follows: (i) control and CSR groups received vehicle (distilled water); (ii) positive control received diazepam (10 mg/kg); three experimental groups were given JSO at a dose of 50 (low), 100 (medium) and 200 (high) mg/kg. (The dosage of JSO administered in this study was determined to be both safe and effective based on prior chronic toxicity testing). All animal experiments complied with the 3R principles (Replacement, Reduction, Refinement) and received approval from the Institutional Animal Care and Use Committee of the Institute of Medicinal Plant Development, Chinese Academy of Medical Sciences (Approval No. SLXD-20240409021).

### 2.3. Experimental Equipment

The major instruments used in this study included a rotating drum sleep deprivation apparatus, an open field assay chamber, a light–dark box assay chamber, and a forced swim assay analyzer (all from Beijing Kangshen Yiyou Technology Co., Ltd., Beijing, China). Additionally, elevated plus maze video analysis was conducted using Any-Maze software (Version 7.15, USA).

### 2.4. Establishment of CSD Model and Behavioral Testing

Mice in the model group, diazepam group, and JSO treatment groups (low, medium, and high dose group) were subjected to CSD stress using a sleep deprivation apparatus consisting of a rotating drum and a computer control system. Based on preliminary optimization by our research group, the following conditions were set: rotation speed (1 r/min), one rotation per cycle, rest period (2 min), and a random rotation direction. Prior to the formal induction of CSD, all groups, except the control group, underwent a 3-day acclimatization period to the rotating drum, with exposure times gradually increasing (3 h/d, 6 h/d, and 8 h/d, respectively). Following acclimatization, model and treatment group mice were subjected to CSD under the preset parameters, with a total rotation time of 8 h/day, continuing until the end of the 3-week modeling duration.

**Figure 1:**
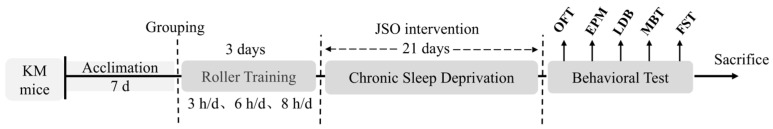
Experimental diagram. (OFT: Open field test; EPM: elevated plus maze test; LDB: light–dark test; MBT: marble burying test; FST: forced swimming test).

Behavioral testing commenced the day after the CSD period. Anxiety-related behaviors were assessed using the open field test (OFT), elevated plus maze test (EPM), light–dark box test (LDT), along with the marble burying and forced swimming test, with one assay conducted per day. Data obtained from these tests were used for subsequent statistical analysis. During the experiment, body weight was measured weekly, with the initial weight recorded prior to the rotarod training. All datasets were incorporated into the final statistical analysis (all as shown in Figure 1).

### 2.5. Behavioral Testing Methods

#### 2.5.1. Open Field Test

Prior to the assay, mice were permitted to acclimatize to the testing room to minimize environmental stress. The experiment took place in a black chamber measuring 40 × 40 cm, which was fitted out with a real-time analysis system that operates on a computer and a camera designed for tracking behavior. Each trial had a duration of 5 min. The primary evaluation parameters were the following: (1) the movement distance in the central zone; (2) the movement time in the central zone. Mice exhibiting anxiety-like behavior typically show reduced exploration and activity in the central zone.

#### 2.5.2. Elevated Plus Maze Test

This assay was designed to measure anxiety-like behaviors by exploiting the innate conflict between an animal’s exploratory drive and its aversion to open, elevated spaces [21,22]. The test duration was 5 min, and the following metrices were recorded: (1) percentage of time spent in the open arms (OT%); (2) percentage of entries into the open arms (OE%). Mice displaying anxiety-like behaviors typically exhibit reduced OT% and OE%, indicating heightened anxiety.

#### 2.5.3. Light–Dark Test

Based on rodents’ natural preference for darkened spaces rather than bright areas, the LDT is commonly used to assess anxiolytic drug effects and screen potential therapeutic compounds. During the test, mice experience a conflict between their natural inclination to remain in the dark compartment and their exploratory drive to investigate the light compartment, thereby eliciting anxiety-like behavior. The test duration was set to 10 min, with two primary evaluation parameters: (1) time spent in the lighting chamber (seconds) and (2) number of transitions among chambers. Anxious mice typically exhibit reduced time spent in the lighting chamber and fewer transitions among chambers, indicating heightened anxiety levels.

#### 2.5.4. Marble Burying Test

This assay was conducted to access repetitive and anxiety-related behaviors in mice. The evaluation included two distinct phases. (1) Adaptation Phase: To minimize neophobia induced by the novel objects (marbles), mice were acclimated in a cage containing fresh bedding and marbles for 20 min prior to the formal test. (2) Testing Phase: The test lasted for 20 min. A marble was deemed buried when at least two-thirds of its height was concealed by corncob bedding. The experimental setup included standard glass marbles (approximately 14 mm in diameter), arranged in a 4 × 5 grid (20 marbles in total). The corncob bedding depth was maintained at approximately 3–5 cm. The testing chamber measures 38 cm (length) × 27 cm (width) × 17 cm (height) and was covered with a transparent acrylic lid to prevent escape. All experiments were conducted under blinded conditions to minimize experimental bias.

#### 2.5.5. Forced Swimming Test

This behavioral paradigm is frequently utilized in rodent studies to evaluate depression-related phenotypes [23,24]. The experimental setup consisted of a cylindrical chamber (18 cm diameter × 20 cm height) filled with 12 cm of water maintained at 23–25 °C. Individual mice were sequentially introduced into this apparatus to ensure proper thermal regulation and prevent hypothermic conditions. Following an initial 2 min acclimation period, the test commenced, and the system automatically logged the total duration of immobility for the subsequent 4 min. Immobility was defined as the state in which the mouse exhibited minimal movement, making only slight limb motions required to keep its head over water, without actively struggling or attempting to escape.

### 2.6. 16S rRNA Microbial Diversity Sequencing

To characterize the intestinal microbial community structure in murine models, 16S rRNA gene sequencing was employed. The procedures for the experiment were conducted as detailed below: Fresh fecal specimens were collected under aseptic conditions, immediately placed in sterile centrifuge tubes, flash-frozen using liquid nitrogen, and stored at −80 °C for subsequent analysis. Genomic DNA was isolated using a standardized commercial extraction kit. The V3-V4 hypervariable domains of bacterial 16S rRNA genes were subsequently amplified by the polymerase chain reaction (PCR) employing the 338F/806R primer set. Amplification products were then purified and their concentration determined using the QuantiFluor™-ST Blue Fluorescence Quantification System (Promega, Madison, WI, USA). Following equimolar pooling of samples according to quantitative measurements, high-throughput sequencing was conducted on the Illumina PE250 platform (Illumina, San Diego, CA, USA). The resulting sequence data were processed using bioinformatics pipelines, wherein sequences with ≥97% similarity were clustered into operational taxonomic units (OTUs). Taxonomic classification was performed to characterize microbial composition and community structure across multiple hierarchical levels. To assess microbial diversity, alpha diversity metrics (Chao1 for richness, Shannon for diversity) were calculated. Comparative analysis of community structures between groups was conducted through beta diversity measures, with results visualized via non-metric multidimensional scaling (NMDS) and principal coordinate analysis (PCoA). Differential species assessment at the genus level was conducted utilizing the Wilcoxon rank-sum test. This study included fecal samples from three experimental groups: control, model, and JSO-Z (medium-dose treatment). From each cohort, eight biological replicates were randomly chosen, collecting approximately 1 g of fecal material per mouse (equivalent to 2–3 pellets per animal).

### 2.7. Western Blot (WB) Analysis

Western blot analysis was performed to quantify protein expression of key signaling molecules (p-mTOR/mTOR, p-PI3K/PI3K, p-AKT/AKT), apoptosis regulators (BAX/BCL-2), inflammasome components (NLRP3, ASC, Caspase-1), cytokines IL-6 and BDNF in murine cortical and hippocampal tissues. Frozen tissue samples (−80 °C preservation) were homogenized in RIPA buffer supplemented with protease/phosphatase inhibitors, using a tissue weight-adjusted volume. After 30 min of ice-cold lysis, samples were centrifuged at 12,000× *g* for 30 min to obtain clarified supernatants. Protein concentrations were normalized across samples using BCA assay measurements. Following SDS-PAGE separation, proteins were electrophoretically transferred to PVDF membranes. Membranes were then incubated with blocking solution prior to immunodetection. Primary antibody incubations were carried out at 4 °C with primary antibodies targeting p-mTOR/mTOR (1:2000, 1:6000), p-PI3K/PI3K (1:1000, 1:1000), p-AKT/AKT (1:2000, 1:1000), BAX/BCL-2 (1:1000, 1:1000), NLRP3 (1:1000), ASC (1:2000), Caspase-1 (1:1000), IL-6 (1:2000), BDNF (1:1000), and GAPDH (1:20,000) as a loading control. Following primary antibody incubation, three 10 min TBST washes were performed. Membranes were then incubated with HRP-linked secondary antibodies (1:5000 dilution) for 60 min at ambient temperature with constant agitation, followed by three subsequent TBST rinses. For chemiluminescent detection, BeyoECL Moon reagent (solutions A and B) was mixed at a 1:1 ratio, vortexed for 30 s, and protected from light. The washed membranes were placed in a clean glass dish, evenly coated with the chemiluminescent reagent, and incubated for 2–3 min at ambient temperature. A chemiluminescence imaging system was employed to visually detect protein bands, and the raw data were collected for subsequent analysis. Relative protein expression was quantified by measuring band intensities in ImageJ software (Version 1.8.0), normalizing all values to the control group for comparative analysis.

### 2.8. Statistical Analysis

All statistical analyses were performed utilizing SPSS 26.0 application. Continuous variables are expressed as mean ± SEM (standard error of the mean). Data distribution normality was evaluated using the Shapiro–Wilk test. For data with a normal distribution, comparisons between two groups were conducted with Student’s *t*-test. Prior to group comparisons, variance homogeneity was assessed via Levene’s test. For data meeting both normality and homoscedasticity assumptions, post hoc LSD analysis was performed. Heterogeneous variances prompted Dunnett’s T3 implementation. Non-parametric alternatives were employed for non-normal distributions, with the statistical significance threshold set at *p* < 0.05.

## 3. Results

### 3.1. Effect of JSO on Body Weight in CSD-Stressed Mice

**Figure 2:**
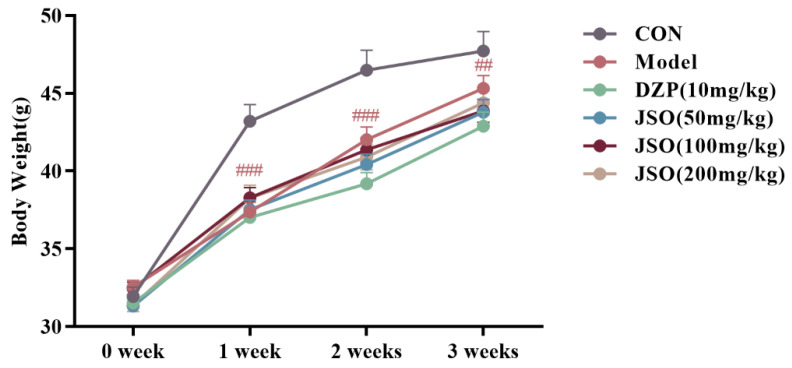
Effect of JSO on body weight in CSD-stressed mice. (*n* = 10–12, mean ± SEM, ## *p* < 0.01, ### *p* < 0.001 vs. the control group. CON: the control group, DZP: the diazepam group, JSO: the *Cichorium intybus* L. Oligo-polysaccharides group, the same in subsequent figures).

Figure 2 illustrates that following 3 weeks of CSD stress, the model group exhibited drastically reduced body mass relative to controls at all time points during weeks 1, 2, and 3 (*p* < 0.001, *p* < 0.01, respectively). However, no statistically significant differences in body weight were observed between the JSO-treated groups and the model group.

### 3.2. Behavior Assessment

#### 3.2.1. Effect of JSO on the OFT in CSD-Stressed Mice

**Figure 3:**
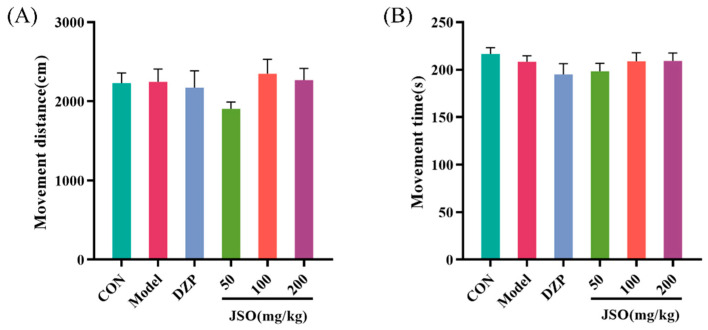
Effect of JSO on OFT performance in CSD-stressed mice. (*n* = 10–12, mean ± SEM, evaluation metrics—(**A**): movement distance/cm; (**B**): movement time/s).

After 3 weeks of CSD stress, locomotor activity analysis revealed no significant differences between model and control groups in either total distance moved (Figure 3A) or cumulative movement duration (Figure 3B), indicating that CSD exposure did not affect spontaneous motor behavior in mice.

#### 3.2.2. Effect of JSO on the EPM in CSD-Stressed Mice

**Figure 4:**
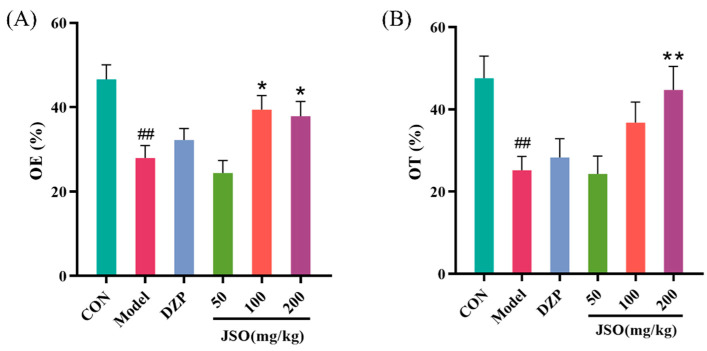
Effect of JSO on the EPM in CSD-stressed mice. (*n* = 10–12, mean ± SEM, evaluation metrics—(**A**) OE%: percentage of entries into open arms; (**B**) OT%: percentage of time spent in open arms. ## *p* < 0.01 vs. control group; * *p* < 0.05, ** *p* < 0.01 vs. model group).

As illustrated in Figure 4A,B, after 3 weeks of CSD stress, the model group demonstrated a marked decrease in both the OE% and the OT% compared to the control group (*p* < 0.01, *p* < 0.01, respectively). By contrast, JSO (200 mg/kg) significantly increased both OE% and OT% compared to the model group (*p* < 0.05, *p* < 0.01, respectively), while JSO (100 mg/kg) greatly increased OT% (*p* < 0.05). These findings suggest that JSO intervention enhances open-arm exploration in CSD-stressed mice, as evidenced by open-arm entries and prolonged time spent in open-arm areas.

#### 3.2.3. Effect of JSO on the LDT in CSD-Stressed Mice

**Figure 5:**
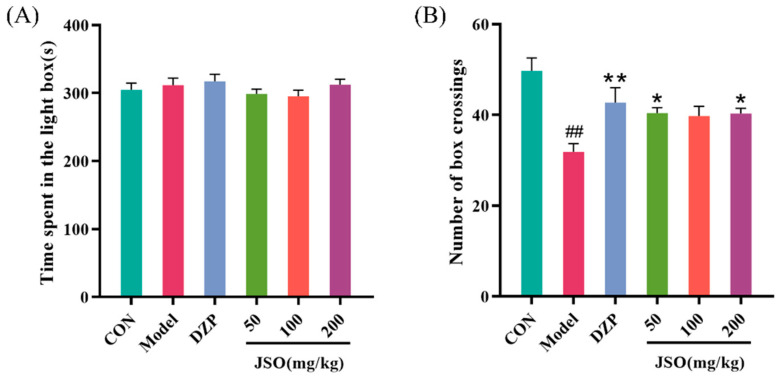
Effect of JSO on the LDT in CSD-stressed mice. (*n* = 10–12, mean ± SEM, evaluation metrics—(**A**) time spent in the light chamber; (**B**) number of transitions. ## *p* < 0.01 vs. control group; * *p* < 0.05, ** *p* < 0.01 vs. model group).

As shown in Figure 5A,B, following a duration of 3 weeks under CSD stress, the model group exhibited a considerable reduction in the number of transitions between compartments in comparison to the control group (*p* < 0.05). Conversely, the DZP (10 mg/kg) group and the JSO (50 mg/kg, 200 mg/kg) groups demonstrated an expanded number of transitions in CSD-stressed mice in comparison to the model group (*p* < 0.01, *p* < 0.05, *p* < 0.05, respectively).

#### 3.2.4. Effects of JSO on the MBT in CSD-Stressed Mice

**Figure 6:**
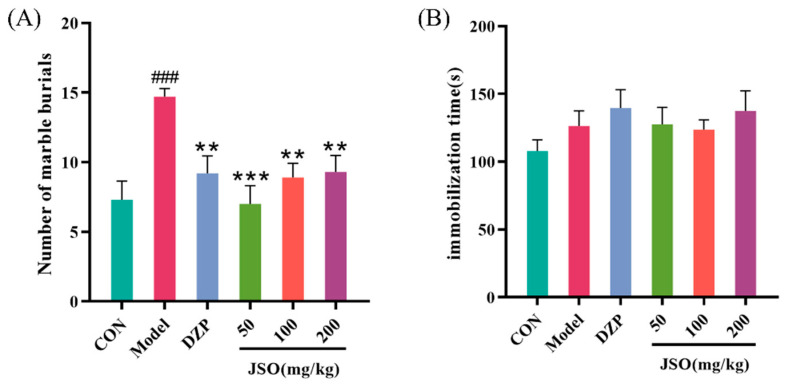
Effect of JSO on the MBT and FST in CSD-stressed mice. (*n* = 10–12, mean ± SEM; (**A**) number of marbles buried; (**B**) immobility time (FST), ### *p* < 0.001 vs. control group; ** *p* < 0.01, *** *p* < 0.001 vs. model group).

Following CSD stress, relative to controls, the model group demonstrated significantly increased marble-burying behavior (*p* < 0.001; Figure 6A). In contrast, the DZP (10 mg/kg) group and the JSO-administered (50 mg/kg, 100 mg/kg, 200 mg/kg) groups had a significantly decreased number of buried items (Figure 6A) in mice that experienced CSD stress when contrasted with the model group (*p* < 0.01, *p* < 0.001, *p* < 0.01, *p* < 0.01, respectively).

#### 3.2.5. Effect of JSO on the FST in CSD-Stressed Mice

After 3 weeks of CSD stress, no markedly significant differences were exhibited between the groups (Figure 6B), suggesting that male KM mice did not develop depression-like behaviors in response to CSD stress.

### 3.3. Effect of JSO on the Gut Microbiota Diversity in CSD-Stressed Mice

#### 3.3.1. Operational Taxonomic Units (OTU) Analysis

**Figure 7:**
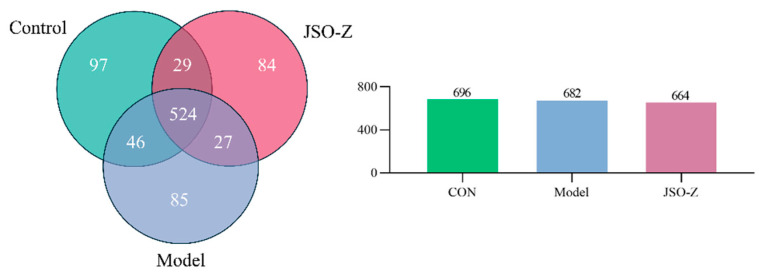
Venn diagram of operational taxonomic units (OTU) among experimental groups. (JSO-Z represents the medium-dose JSO administration group, the same in subsequent figures).

The Venn diagram (Figure 7), generated through OTU analysis, illustrates the unique and overlapping OTUs among the different sample groups, offering a visual representation of microbial community composition at the OTU level. Comparative analysis revealed 524 operational taxonomic units (OTUs) common to all experimental groups: controls, CSD model, and JSO-treated animals. Additionally, the blank control, model, and JSO intervention groups exhibited 97, 84, and 85 unique OTUs, respectively.

#### 3.3.2. Biodiversity Analysis of Gut Microbiota

**Figure 8:**
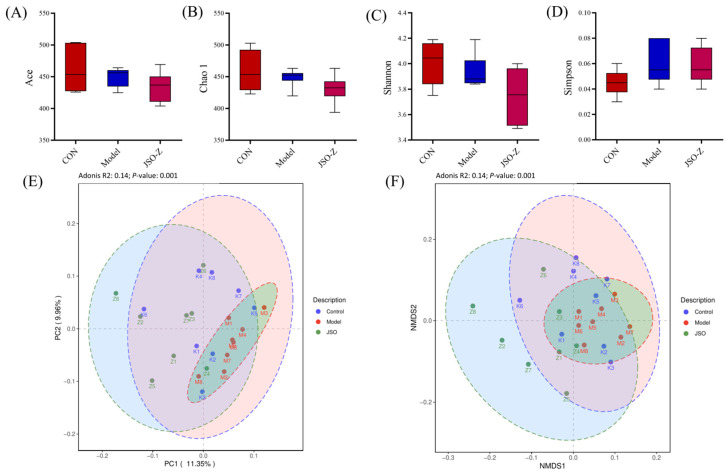
Biodiversity analysis of gut microbiota. (*n* = 8, mean ± SEM; (**A**) ACE; (**B**) Chao1; (**C**) Shannon; (**D**) Simpson; (**E**) PcoA, *p* = 0.001, R^2^ = 0.14; (**F**) NMDS, *p* = 0.001, R^2^ = 0.14).

Alpha diversity analysis was conducted to assess species richness and diversity across all samples (Figure 8A–D). Alpha diversity analysis revealed non-significant reductions in both ACE and Chao1 indices for the model group relative to blank controls, indicating a potential (though statistically insignificant) decline in microbial richness. Similarly, no significant intergroup differences were observed in Shannon or Simpson diversity indices. To further evaluate intergroup differences, beta diversity analysis was conducted (Figure 8E,F). Principal coordinate analysis (PCoA, Figure 8E) further confirmed these differences (*p* = 0.001, R^2^ = 0.14). Non-metric multidimensional scaling (NMDS, Figure 8F) analysis demonstrated significant differences among groups (*p* = 0.001, R^2^ = 0.14), indicating that intergroup variation was significantly greater than intragroup variation. These findings suggest that the gut microbiota composition varied significantly among the sample groups.

#### 3.3.3. Analysis of Gut Microbiota Composition at the Phylum and Genus Levels

**Figure 9:**
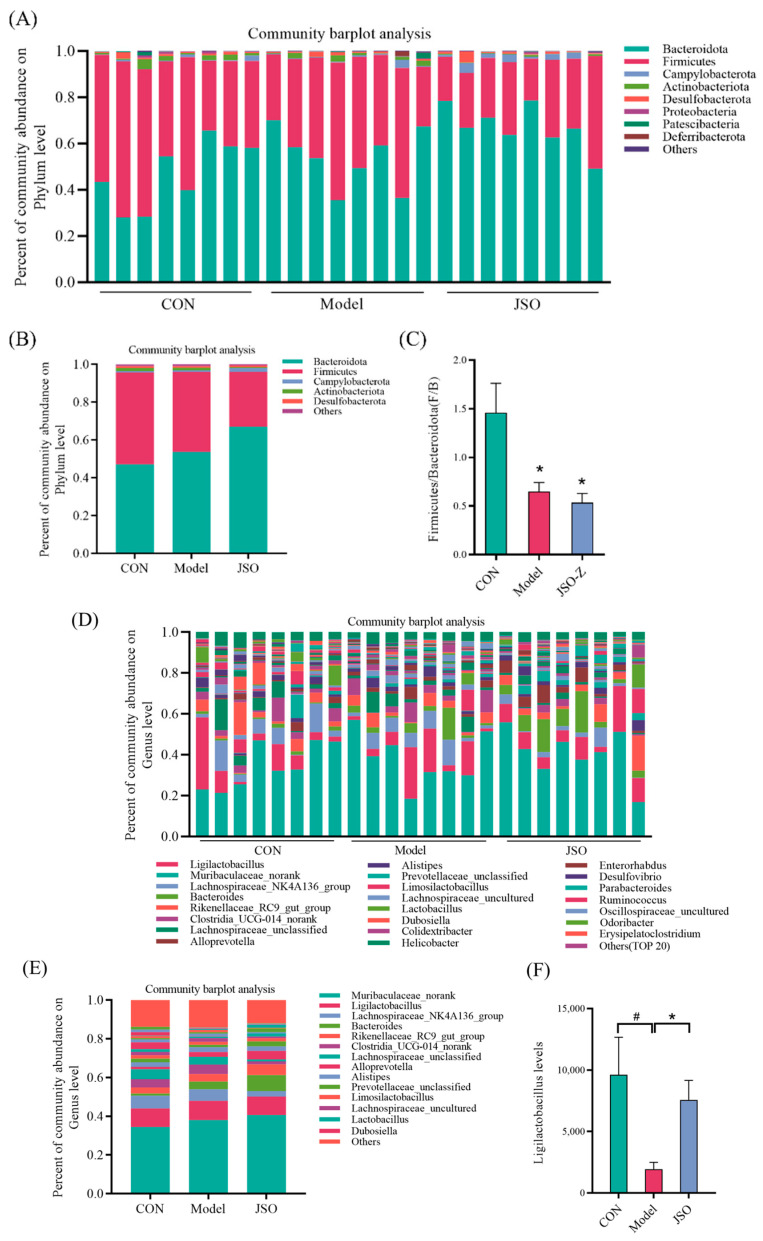
Gut microbial composition at the phylum and genus levels. (**A**,**B**) Percent of community abundance on Phylum level; (**C**) the Firmicutes-to-Bacteroidetes (F/B) ratio in the mice gut microbiota; (**D**,**E**) percent of community abundance on genus level; (**F**) the *Ligilactobacillus* levels. (# *p* < 0.05 vs. control group; * *p* < 0.05, vs. JSO group).

Displayed in Figure 9A,B is the phylum-level composition of gut microbes, illustrating individual samples as well as group averages for the blank control, model, and JSO treatment groups. The five predominant bacterial phyla across all samples were *Bacteroidetes*, *Firmicutes*, *Campylobacterota*, *Actinobacteriota*, and *Desulfobacterota*. The ratio of Firmicutes to Bacteroidetes (F/B) (see Figure 9C) was notably reduced, compared to control, in the model group (*p* < 0.05). Following JSO intervention, the relative abundance of *Campylobacterota* was elevated, whereas *Actinobacteriota* decreased. At the genus level (Figure 9D,E), the top five genera were *Muribaculaceae* norank, *Ligilactobacillus*, *Lachnospiraceae NK4A136* group, *Bacteroides*, and *Rikenellaceae RC9* gut group. The model group had a reduced proportion of *Lachnospiraceae NK4A136* group when in comparison to the control group, along with a notable decrease in *Ligilactobacillus* abundance (*p* < 0.05). However, JSO intervention was linked to a raised relative abundance of the *Rikenellaceae RC9* gut group (*Bacteroidetes*) and a significant enhancement in *Ligilactobacillus* levels (*p* < 0.05).

#### 3.3.4. Differential Microbial Genera Analysis in JSO-Treated Gut

**Figure 10:**
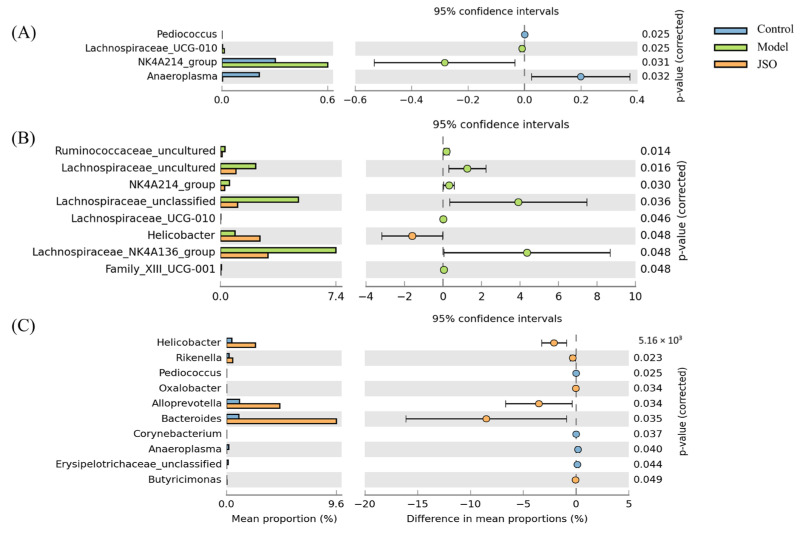
Differential species analysis based on Wilcoxon rank-sum test. (**A**)—control group vs. model group; (**B**)—model group vs. JSO medium-dose group; (**C**)—control group vs. JSO medium-dose group.

To evaluate JSO’s effects on gut microbial composition, differential genus-level species were analyzed using the Wilcoxon rank-sum test. As illustrated in Figure 10, the model group demonstrated a notable rise in the abundance of the unclassified genus NK4A214group (*Firmicutes*) and a considerable decline *Anaeroplasma* (*Anaeroplasmataceae*) in comparison to the control group. Following JSO intervention, there was a marked increase in the abundance of *Helicobacter*, *Alloprevotella* (*Prevotellaceae*), and *Bacteroides*. Additionally, in comparison with the model group, JSO treatment group revealed a substantial reduction in the abundance of *Lachnospiraceae* uncultured, *Lachnospiraceae* unclassified, and *LachnospiraceaeNK4A136group* (*Lachnospiraceae*), while *Helicobacter* abundance significantly increased.

#### 3.3.5. Functional Annotation Analysis of KEGG Metabolic Pathways

**Figure 11:**
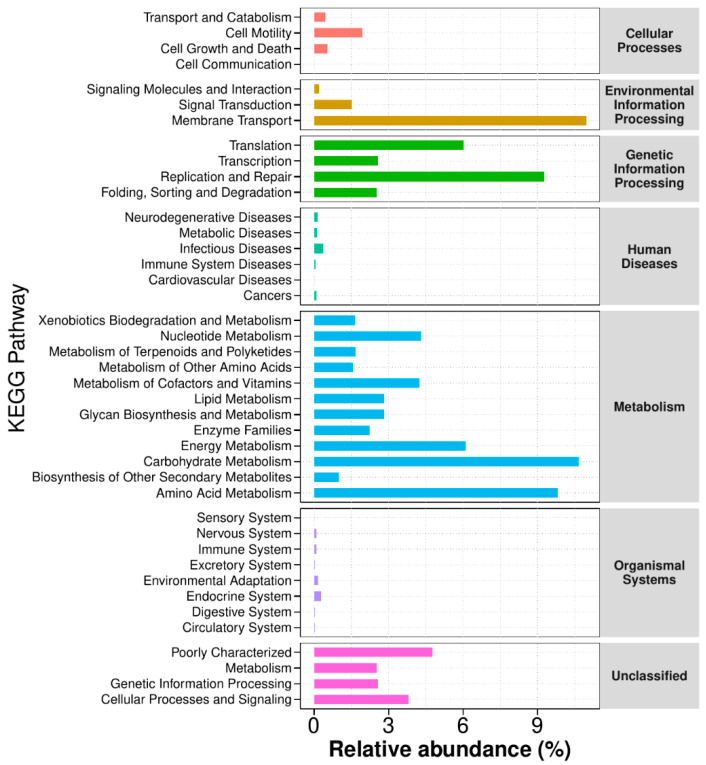
Functional annotation analysis of KEGG metabolic pathways. (Data analysis was conducted using the control and model groups).

Analysis of KEGG metabolic pathways through functional annotation (Figure 11) indicated significant variations in microbial functional genes across various sample groups. This analysis provides valuable insights into the metabolic functional changes of microbial communities in response to environmental factors. Differentially abundant microbial communities were enriched in multiple functional categories related to metabolism, genetic information processing, cellular processes and signaling, and human diseases, highlighting the potential influence in host–microbe interactions and disease pathophysiology. Microbial-mediated metabolic pathways (carbohydrate, amino acid, and lipid metabolism) were prominently identified, highlighting the microbiota’s central function in regulating host metabolic homeostasis. Nevertheless, the exact mechanisms driving these microbial-induced metabolic changes require further exploration.

### 3.4. Effect of JSO on Cortical Pathway-Related Proteins in CSD-Stressed Mice

#### 3.4.1. Effect of JSO on the Expression of PI3K/AKT/mTOR Pathway Proteins

**Figure 12:**
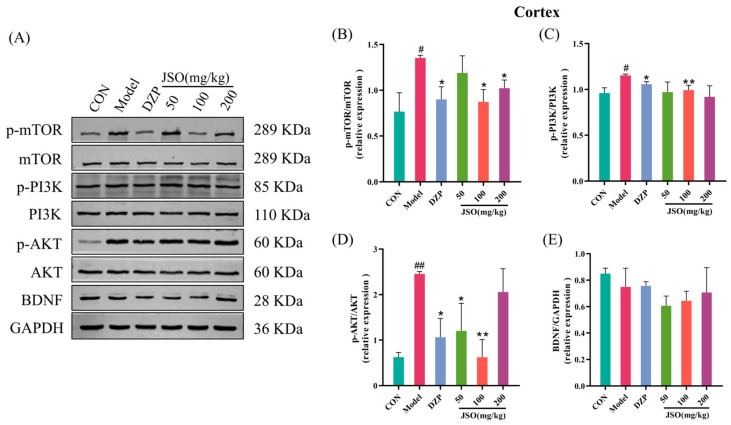
Effect of JSO on PI3K/AKT/mTOR pathway-related proteins in the cortex of CSD-stressed mice. (**A**) Western blot images. (**B**–**D**) The ratio of p-mTOR/mTOR, p-PI3K/PI3K, and p-AKT/AKT proteins. (**E**) The expression of BDNF protein. CON: the control group, DZP: the diazepam group, JSO: the *Cichorium intybus* L. Oligo-polysaccharides group, the same in subsequent figures. (*n* = 3–4, mean ± SEM; vs. the control group, # *p* < 0.05, ## *p* < 0.01; vs. the model group, * *p* < 0.05, ** *p* < 0.01).

As illustrated in Figure 12B–D, the model group exhibited a notable elevation in the relative expression of p-mTOR/mTOR, p-PI3K/PI3K, and p-AKT/AKT proteins when in comparison with the control group (*p* < 0.05, *p* < 0.05, *p* < 0.01, respectively). Conversely, administration of DZP (1.5 mg/kg) and JSO (100 mg/kg, 200 mg/kg) led to a significant reduction in p-mTOR/mTOR expression contrasted with the model group (*p* < 0.05, *p* < 0.05). Likewise, the p-PI3K/PI3K ratio was significantly decreased by both DZP (1.5 mg/kg) and JSO (100 mg/kg) (*p* < 0.05, *p* < 0.01). Furthermore, DZP (1.5 mg/kg) along with JSO (50 mg/kg, 100 mg/kg) greatly diminished the p-AKT/AKT ratio (*p* < 0.05, *p* < 0.05, *p* < 0.01). BDNF protein expression showed no statistically significant variation across the experimental groups (Figure 12E). These results reveal that JSO intervention effectively downregulated the P13K/AKT/mTOR signaling pathway, potentially contributing to its therapeutic effects.

#### 3.4.2. Effect of JSO on p-NF-κB/NF-κB and BAX/BCL-2 Proteins Expression in the Cortex

**Figure 13:**
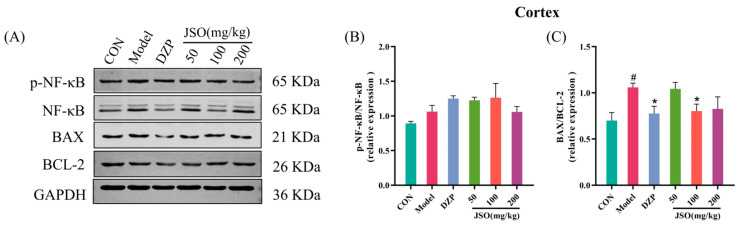
Effect of JSO on p-NF-κB/NF-κB and BAX/BCL-2 Proteins in the cortex of CSD-stressed mice. (**A**) Western blot images. (**B**,**C**) The ratio of p-NF-κB/NF-κB and BAX/BCL-2 proteins. (*n* = 3–4, mean ± SEM; # *p* < 0.05 vs. the control group; * *p* < 0.05 vs. the model group).

As demonstrated in Figure 13C, the ratio of BAX to BCL-2 proteins was significantly increased in the cortex of the model group when contrasted with the control (*p* < 0.05). Nonetheless, administering DZP (1.5 mg/kg) and JSO (100 mg/kg) led to a marked reduction in the BAX/BCL-2 ratio relative to the model (*p* < 0.05). There were no notable alterations in the ratio of p-NF-κB/NF-κB protein expression (Figure 13B). These findings indicate that JSO intervention may exert neuroprotective effects by modulating the apoptotic signaling pathways in the cortex of CSD-stressed mice.

### 3.5. Effect of JSO on Hippocampal Pathway-Related Proteins in CSD-Stressed Mice

#### 3.5.1. Effect of JSO on PI3K/AKT/mTOR Pathway and BDNF Expression in the Hippocampus

**Figure 14:**
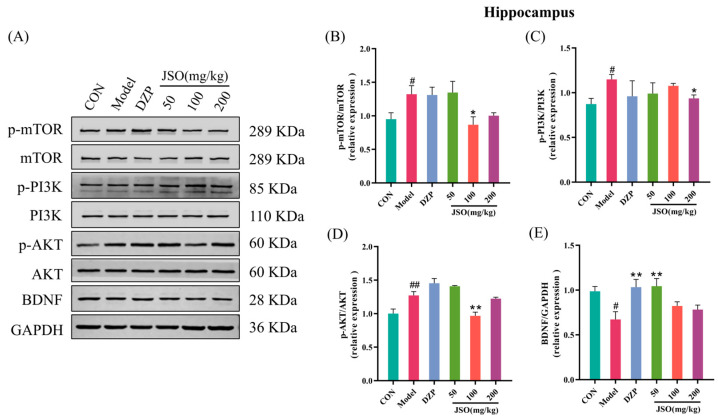
Effect of JSO on proteins associated with the PI3K/AKT/mTOR pathway in the hippocampus of CSD-stressed mice. (**A**) Western blot images. (**B**–**D**) The ratio of p-mTOR/mTOR, p-PI3K/PI3K, and p-AKT/AKT proteins. (**E**) The expression of BDNF protein. (*n* = 3–4, mean ± SEM; # *p* < 0.05, ## *p* < 0.01 vs. the control group; * *p* < 0.05, ** *p* < 0.01 vs. the model group).

The model group exhibited an essential increase of p-mTOR/mTOR (Figure 14B), p-PI3K/PI3K (Figure 14C), and p-AKT/AKT (Figure 14D) expression ratios in the hippocampus compared to the control group (*p* < 0.05, *p* < 0.05, *p* < 0.01, respectively). Furthermore, the expression levels of BDNF were considerably decreased in the model group (*p* < 0.05) as indicated in Figure 14E. In contrast, treatment with JSO (100 mg/kg) resulted in an appreciable reduction in the p-mTOR/mTOR and p-AKT/AKT protein ratios ratio versus the model group (*p* < 0.05, *p* < 0.01), while JSO (200 mg/kg) significantly decreased the p-PI3K/PI3K protein ratio (*p* < 0.05). Furthermore, DZP (1.5 mg/kg) and JSO (50 mg/kg) significantly elevated the hippocampal BDNF protein expression (*p* < 0.01, *p* < 0.01, respectively). The findings suggest JSO modulates the PI3K/AKT/mTOR signaling pathway and promotes BDNF expression, potentially contributing to its neuroprotective effects in CSD-stressed mice.

#### 3.5.2. Effects of JSO on p-NF-κB/NF-κB and BAX/BCL-2 Proteins Expression in the Hippocampus

**Figure 15:**
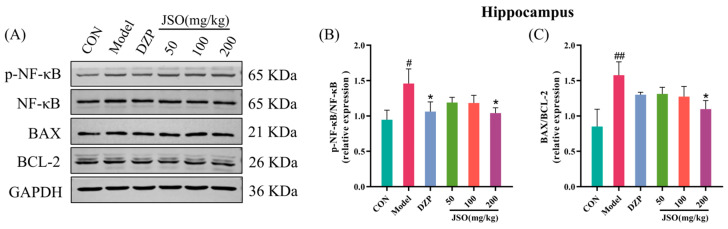
Effect of JSO on p-NF-κB/NF-κB and BAX/BCL-2 proteins in the hippocampus of CSD-stressed mice. (**A**) Western blot images. (**B**,**C**) The ratio of p-NF-κB/NF-κB and BAX/BCL-2 proteins. (*n* = 3–4, mean ± SEM; # *p* < 0.05, ## *p* < 0.01 vs. the control group; * *p* < 0.05 vs. the model group).

The model group demonstrated a substantial increase in the p-NF-κB/NF-κB (Figure 15B) and BAX/BCL-2 (Figure 15C) protein ratios in the hippocampus contrasted with the control group (*p* < 0.05, *p* < 0.01, respectively). In contrast, DZP (1.5 mg/kg) treatment significantly reduced the p-NF-κB/NF-κB protein ratio relative to the model group (*p* < 0.05). Similarly, treatment with JSO (200 mg/kg) significantly reduced both the p-NF-κB/NF-κB and BAX/BCL-2 protein ratios (*p* < 0.05). These findings suggest that JSO exerts neuroprotective effects by modulating NF-κB signaling pathways and proteins related with apoptosis in the hippocampus of mice exposed to chronic stress.

#### 3.5.3. Effects of JSO on Hippocampal Neuroinflammation-Associated Proteins

**Figure 16:**
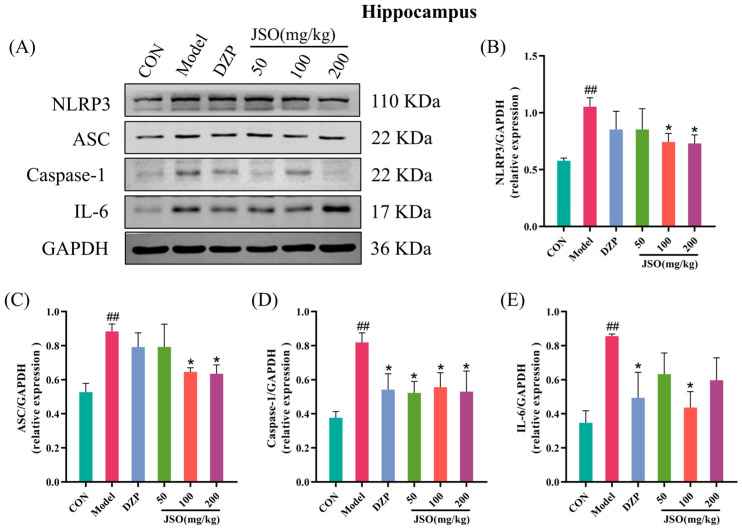
Effects of JSO on hippocampal neuroinflammation-associated proteins of CSD-stressed mice. (**A**) Western blot images. (**B**–**E**) The expression of NLRP3, ASC, and Caspase-1 and IL-6 proteins. (*n* = 3–4, mean ± SEM; ## *p* < 0.01 vs. the control group; * *p* < 0.05 compared to the model group).

Relative to controls, hippocampal expression of NLRP3 (Figure 16B), ASC (Figure 16C), Caspase-1 (Figure 16D), and IL-6 (Figure 16E) was significantly upregulated in the model group (all *p* < 0.01, respectively). In contrast, DZP (1.5 mg/kg) greatly reduced the expression of Caspase-1 and IL-6 proteins. The JSO (100 mg/kg, 200 mg/kg) groups substantially decreased the NLRP3, ASC, and Caspase-1 proteins expression (*p* < 0.05, *p* < 0.05). Moreover, the JSO (50 mg/kg) dose group substantially downregulated Caspase-1 protein (*p* < 0.05), while the JSO (100 mg/kg) dose group significantly downregulated IL-6 expression (*p* < 0.05). Collectively, these findings demonstrate that JSO exerts anti-neuroinflammatory effects in CSD-exposed mice through hippocampal regulation of both NLRP3 inflammasome activation and IL-6 production.

## 4. Discussion

This study demonstrates that JSO intervention effectively alleviates anxiety-like behaviors that result from chronic sleep deprivation (CSD) stress in KM mice. The underlying mechanisms involve JSO-mediated modulation of gut microbiota balance, suppression of aberrant activation of the BDNF-PI3K/AKT/mTOR and BAX/BCL-2 pathways in the cortex and hippocampus, and enhancement of BDNF protein expression. These molecular effects contribute to key physiological processes, including neuronal survival and synaptic plasticity. Furthermore, JSO reversed the dysregulated levels of inflammatory factors such as NLRP3, ASC, Caspase-1, and IL-6, suggesting that JSO inhibits NLRP3 inflammasome-mediated neuroinflammation in the hippocampus, which further supports its anxiolytic effects. These findings additionally reinforce the potential of JSO as a treatment option for anxiety disorders.

The CSD model (rotating drum method) employs a motorized device to regulate rotation time and speed, leading to reduced total sleep time and increased sleep fragmentation, which ultimately lowers sleep quality and induces sleep deprivation. This model effectively simulates human anxiety-like behaviors associated with sleep disturbances and social stress-induced insomnia. In order to thoroughly assess anxiety-related behaviors, a variety of behavioral assessments were conducted, such as the open field test (OFT), elevated plus maze (EPM), light–dark transition test (LDT), marble burying test (MBT), and forced swim test (FST). Behavioral analysis revealed that JSO intervention significantly increased the OE% and the OT% in the EPM, indicating that JSO reduced fear and enhanced exploratory behavior in CSD-stressed mice. Additionally, JSO significantly increased the number of transitions in the LDT, promoting exploration of the light chamber, and markedly reduced marble-burying behavior in the MBT, suggesting attenuation of defensive response. To evaluate possible depressive-like behaviors, a forced swim test was performed. The findings indicated no notable differences in the duration of immobility between the groups, implying that CSD stress did not provoke depressive-like behaviors in the mice.

Recent research highlights the gut microbiota’s critical influence on host physiology, particularly through the bidirectional gut–brain axis communication with the CNS. This crosstalk regulates neuroendocrine pathways and influences affective behaviors. Notably, microbial dysbiosis has been consistently linked to anxiety-like phenotypes in animal models [25,26]. Among the dominant microbial phyla, *Firmicutes* and *Bacteroidetes* are key indicators of host health, as their ratio is closely associated with physiological hemostasis. Metabolites, including short-chain fatty acids (SCFAs) and precursors of neurotransmitters, are produced by these microbial communities and can directly influence brain function via the gut–brain axis [27,28]. In this study, high-throughput 16S rRNA sequencing revealed that CSD-stressed mice exhibited a significantly lower *Firmicutes*/*Bacteroidetes* (F/B) ratio compared to the control group, a finding in line with previous research demonstrating stress-induced reductions in the F/B ratio [29,30]. Additionally, the abundance of the *Ligilactobacillus* genus was significantly lowered in CSD-stressed mice. *Ligilactobacillus*, a subgenus of *Lactobacillus*, is a Gram-positive bacterial group with known probiotic properties. Certain strains, such as *Ligilactobacillus salivarius*, have been reported to regulate intestinal metabolism and immune function, with their abundance being closely linked to the host health [31,32]. Notably, JSO intervention significantly increased the relative abundance of *Ligilactobacillus*, further supporting its regulatory effects on the gut microbiota composition. To explore microbial differences at the genus level, Wilcoxon rank-sum tests were performed, revealing significant increases in the abundance of *Bacteroides* and *Alloprevotella* following JSO treatment. *Bacteroides,* one of the dominant genera in human and animal gut microbiota, play a vital role in breaking down dietary fibers and polysaccharides into SCFAs, which serve as energy sources for intestinal epithelial cells and contribute to maintaining intestinal barrier integrity [33,34]. Similarly, *Alloprevotella*, a Gram-negative, anaerobic bacterium belonging to the *Prevotellaceae* family, is renowned for its capacity to degrade complex carbohydrates and proteins while modulating the host immune response [35]. Collectively, these findings suggest that JSO alleviates anxiety-like behaviors in CSD-stressed mice by modulating the gut microbiota composition, restoring the F/B ratio, and promoting beneficial bacterial populations.

Brain-derived neurotropic factor (BDNF) has high levels in multiple brain regions, especially in the hippocampus and prefrontal cortex, which are closely involved in emotion regulation [36,37,38]. By binding to its receptor, TrkB, BDNF enhances neuronal excitability and synaptic plasticity, thereby modulating mood and emotional stability. Decreased BDNF expression has been mechanistically linked to diminished neuronal survival and synaptic plasticity deficits, which collectively promote the emergence of anxiety-related behavioral phenotypes [39,40]. The signaling pathway involving PI3K/AKT/mTOR is essential for several intracellular physiological functions, such as cell growth, regulation of metabolism, and the synthesis of proteins [41,42]. Dysregulation of this pathway can disrupt intracellular homeostasis and impair normal neuronal function. Furthermore, aberrant activation of this pathway contributes to the pathogenesis of anxiety by dysregulating synaptic and neuroplasticity in the cortex and hippocampus [43,44]. Notably, excessive activation of mTOR has been shown to interfere with cellular autophagy [45], leading to the accumulation of neurotoxic proteins within neurons, thereby exacerbating neurodegenerative changes and anxiety symptoms [46,47]. Therefore, BDNF plays a pivotal role in regulating synaptic plasticity, neurodevelopment, neuronal metabolism, and neuroinflammation through the PI3K/AKT/mTOR signaling pathway [48,49,50]. Western blot analysis in this study revealed aberrant activation of the BDNF-PI3K/AKT/mTOR signaling pathway in the cortex and hippocampus of CSD-stressed mice, accompanied by significantly reduced hippocampal BDNF levels. However, JSO intervention suppressed PI3K/AKT/mTOR pathway activity in both brain regions while markedly elevating hippocampal BDNF expression. These findings demonstrate that JSO ameliorates anxiety-like behaviors by attenuating BDNF-mediated hyperactivation of the PI3K/AKT/mTOR signaling pathway.

BAX and BCL-2 are critical proteins involved in the regulation of apoptosis, and their roles in anxiety disorders have garnered increasing attention in recent years. BAX, a pro-apoptotic member of the Bcl-2 protein family, is activated under stress conditions and translocates to the mitochondrial membrane. This leads to increased mitochondrial membrane permeability, cytochrome C release, and subsequent activation of the caspase cascade, ultimately triggering apoptosis [51]. In contrast, BCL-2, an anti-apoptotic protein, maintains mitochondrial membrane stability by inhibiting BAX activity, thereby preventing cytochrome C release and the initiation of apoptosis [52]. Research has demonstrated that chronic stress significantly upregulates BAX expression while downregulating BCL-2 levels in the hippocampus and prefrontal cortex, resulting in increased neuronal apoptosis [53,54]. This dysregulation may play a pivotal role in the pathogenesis and progression of anxiety disorders. Results showed a significant increase in the BAX/BCL-2 ratio in both the hippocampus and cortex of mice, whereas JSO intervention effectively reduced this ratio, attenuating neuronal apoptosis and restoring neural homeostasis.

**Figure 17:**
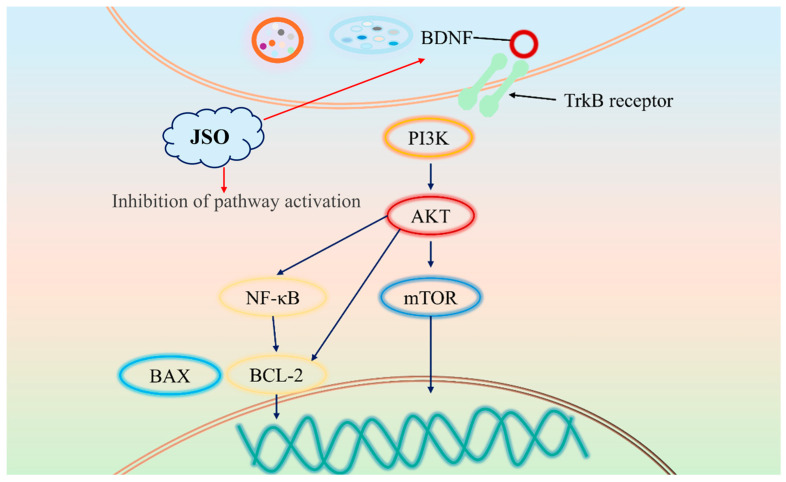
JSO may participate in BDNF-PI3K/AKT/mTOR and BAX/BCL-2 signaling pathways involved in improving anxiety-like behaviors.

Therefore, JSO ameliorates anxiety-like behaviors by upregulating BDNF expression, suppressing hyperactivation of the PI3K/AKT/mTOR signaling pathway, and reducing the BAX/BCL-2 ratio, thereby enhancing neuronal survival and synaptic plasticity. The pathways through which JSO ameliorates anxiety-like behaviors are illustrated in Figure 17.

A mounting volume of evidence indicates a strong correlation between anxiety and neuroinflammation. Neuroinflammation can disrupt neurotransmitter metabolism, impair synaptic plasticity, and compromise neuronal survival, thereby exacerbating anxiety symptoms by activating the hypothalamic–pituitary–adrenal (HPA) axis [55,56,57]. The NLRP3 inflammasome is a multiprotein complex comprising NLRP3, ASC, and Caspase-1, and its activation leads to the maturation and release of pro-inflammatory cytokines like IL-1β and IL-18 [58]. A multitude of studies has shown a robust link between the activation of the NLRP3 inflammasome and behaviors that resemble anxiety [59,60]. Western blot analysis in this study revealed a significant upregulation of NLRP3, ASC, Caspase-1, and IL-6 proteins in the hippocampus of CSD-stressed mice, indicating that CSD stress induces hippocampal neuroinflammation. However, JSO intervention significantly downregulated the expression of these proteins, suggesting that JSO effectively inhibits NLRP3 inflammasome-mediated neuroinflammation in the hippocampus. These results highlight the promise of JSO as a neuroprotective agent in mitigating neuroinflammation-induced anxiety-like behaviors by targeting the NLRP3 inflammasome pathway.

## 5. Conclusions

This study demonstrates that JSO exerts significant anti-anxiety effects by modulating the gut microbiota composition, particularly by enhancing the abundance of *Ligilactobacillus*, *Bacteroides*, and *Alloprevotella*, thereby promoting intestinal homeostasis. Furthermore, JSO regulates the BDNF-PI3K/AKT/mTOR and BAX/BCL-2 signaling pathways, preventing neuronal apoptosis and enhancing synaptic plasticity. Moreover, it effectively suppresses NLRP3 inflammasome-mediated neuroinflammation, further contributing to its anxiolytic properties. These results provide new understanding of the anti-anxiety potential of JSO, highlighting its therapeutic relevance. Future research will concentrate on elucidating the intricate interplay between gut microbiota and neuronal signaling pathways, thereby advancing our understanding of JSO’s role in anxiety management. By elucidating the potential mechanisms underlying the anxiolytic effects of JSO, this study provides a valuable theoretical foundation for its development as a functional food ingredient, dietary supplement, or even a pharmaceutical agent.

## Data Availability

The data presented in this study are available on request from the corresponding author. (The data are not publicly available due to privacy).
